# Identification of urgent gaps in public and policymaker knowledge of heart failure: Results of a global survey

**DOI:** 10.1186/s12889-023-15405-4

**Published:** 2023-05-30

**Authors:** Carolyn S. P. Lam, Ed Harding, Marc Bains, Alex Chin, Naresh Kanumilli, Mark C. Petrie, Paula Pohja-Hutchison, Jiefu Yang, Javed Butler

**Affiliations:** 1 National Heart Centre Singapore, Duke–NUS Medical School, 169857 Singapore, Singapore; 2 The Health Policy Partnership, WC2N 4JS London, UK; 3 The HeartLife Foundation, V5X 1E7 Vancouver, BC Canada; 4 AstraZeneca, Global Medical Affairs, 20878 Gaithersburg, MD USA; 5 Northenden Group Practice, M22 4DH Northenden, Manchester, UK; 6 Institute of Cardiovascular and Medical Sciences, The University Court of the University of Glasgow, G12 8QQ Glasgow, UK; 7 AstraZeneca, Global Corporate Affairs, CB2 8PA Cambridge, UK; 8 Department of Cardiology, Beijing Hospital, National Center of Gerontology China, 100730 Beijing, China; 9 Department of Medicine, University of Mississippi Medical Center, 39216 Jackson, MS USA

**Keywords:** Heart failure, Healthcare, Early diagnosis, Public health

## Abstract

**Background:**

Despite advances in the treatment of heart failure (HF) with reduced ejection fraction, people with HF continue to have a high risk of mortality and hospitalisation. Patients also suffer from poor quality of life, with reduced societal and economic participation. The burden of HF on patients and healthcare systems is extraordinary, yet awareness remains low. This survey was conducted to identify gaps in general public and policymaker knowledge around HF.

**Methods:**

A closed-question web-based survey of the general public and policymakers was conducted between February and October 2020. Study outcomes assessed the participants’ awareness and understanding of HF symptoms, risk factors and mortality, and views around hospital admissions in their country. Responses were collected using multiple-choice questions.

**Results:**

The survey was completed by 26,272 general public respondents in 13 countries and 281 government and public sector policymakers in nine countries. While 99% of general public respondents had heard of HF, their understanding of the condition and its symptoms was poor, and only 6% identified that shortness of breath, fatigue, and leg swelling were the main symptoms of HF. Of policymaker respondents, 14% identified HF as the leading cause of avoidable hospitalisations, and only 4% recognised that ~ 87% of government spending on HF is related to hospitalisations.

**Conclusions:**

Major gaps were identified in the understanding of HF and the burden it places on patients and their caregivers, healthcare systems and society. This study confirms an ongoing need for national policy strategies and investment to raise awareness of the importance of HF prevention, early diagnosis, and implementation of effective treatments to reduce hospitalisations and death.

**Supplementary Information:**

The online version contains supplementary material available at 10.1186/s12889-023-15405-4.

## Background

Heart failure (HF) refers to an inability of the heart to pump blood to the body at a rate commensurate with its needs, or to do so only at the cost of high filling pressures.[1] It manifests as a clinical syndrome with typical symptoms and signs; and is a progressive condition with diverse causes including hypertension, inherited cardiac conditions, valvular heart disease and myocardial infarction (MI).[2] HF is a global public health concern, affecting over 64 million people worldwide.[3] In the USA and Europe, patients with HF account for 1–3% of hospital admissions and the condition is the leading single cause of hospitalisation.[4, 5] Patients over the age of 65 account for 80% of hospitalisations for HF and 90% of HF-related deaths.[6] Population growth and an increasing ageing population are associated with escalating global health problems, including rising prevalence of HF.[7] HF is not just a concern for older people, as it also has a rising incidence and associated mortality in people under 65.[8, 9] The overall cost of HF across the globe was USD 108 billion in 2012,[7] and in the USA alone, medical costs for patients with HF are expected to rise from USD 20.9 billion in 2012 to USD 53.1 billion by 2030.[10] Hospitalisations and inpatient care are the major cost driver in HF, accounting for up to 87% of associated spending.[11] In low- and middle-income regions of the world where HF strikes people at a younger age, there are also increased associated indirect costs, such as lost productivity.[12].

The growing burden of the disease is a challenge for the sustainability of healthcare systems, requiring policymakers to develop and implement coordinated HF strategies. Proven models of prevention and management can improve quality of life as well as reduce mortality and hospitalisations in patients with HF.[13] The prognosis for HF, particularly HF with reduced ejection fraction, has improved over recent decades with advances in medication and device therapy.[14–17] Despite these improvements, there is still a lack of understanding of what HF is, and of its associated risks and healthcare burden.[18, 19] One in five people will develop HF in their lifetime, and approximately half of people diagnosed with HF will die within five years of their diagnosis.[20, 21] These outcomes are worse than most cancers and are comparable to the five-year survival rate for ovarian cancer, leukaemia and myeloma.[22–24] Clinicians and patients are not always aware that the risk of mortality after hospitalisation for HF is as high as for MI or stroke,[25, 26] leading to lack of prioritisation of appropriate care for their worsening HF.

Low awareness and understanding of HF and its associated burden among policymakers, clinicians, patients and the public remains a barrier to the implementation of best practice in prevention, evaluation and treatment.[13] Accordingly, the current survey was conducted among the general public and policymakers to ascertain current levels of HF awareness, and to identify gaps in global understanding of HF and recognition of the burden it places on healthcare systems.

## Methods

### Survey methodology and participants

The study data were obtained through closed-question surveys conducted by YouGov. Members of the general public sitting on YouGov panels, representative of the national population in terms of their age, sex and geographical distribution across regions, were invited randomly by email to participate. No conditions were applied to sample for any specific population demographic. The email contained a unique link to the survey on the YouGov platform, and responses were collected until quotas were met. Participants who indicated that they had been diagnosed by a doctor as having HF were screened out of the survey. As policymakers do not sit on existing panels, policy decision-makers in government and public sector organisations were directly invited by email to complete the survey. Policymaker respondents were elected individuals at the national level or senior members of their staff and were as evenly distributed as possible across the main political parties in a given country based on the current make-up of the government. These may also have included elected mayors or members of the second house depending on the country/system, but a political mix that broadly reflected the current state of the government of that country was maintained. Policymaker respondent email addresses were obtained through government websites, or sourced through methods including social networks, web intercepts, affiliate websites and membership platforms/databases. All respondents were required to visit a secure registration page and opt in before they were eligible to receive survey invitations.

The national quotas were 2000 for number of general public respondents, and 30 for policymaker respondents. The surveys were designed to be completed in 6–8 minutes and were translated into the local language for each country in which respondents were recruited (Table 1). Responses were collected through the use of multiple-choice questions, with the order of the response options randomised among participants. Some questions were presented with Likert scale response options, which were not randomised. Each respondent could only participate in the survey once, and answers could not be amended once submitted.

**Table 1:** **Survey and respondent numbers by country**

Country	Language	General public respondents	Policymaker respondents
Brazil	Brazilian Portuguese	2018	–
Canada	English	2013	30
China	Chinese (simplified)	2025	–
Germany	German	2057	30
Indonesia	Indonesian	–	31
Italy	Italian	2012	32
Japan	Japanese	2012	–
Malaysia	Malay	–	30
Poland	Polish	2011	–
Romania	Romanian	2002	–
Spain	Spanish	2018	33
Taiwan	Chinese (simplified)	2007	30
Thailand	Thai	2009	–
United Kingdom (UK)	English	2047	30
United States of America (USA)	English	2041	35
**Total**		**26,272**	**281**

The first wave of surveys, conducted in Brazil, Canada, China, Germany, Italy, Japan, Spain, the UK and the USA, were obtained between 24^th^ February to 6^th^ March 2020. The second wave of surveys, conducted in Malaysia, Poland, Romania and Thailand, were obtained between 6^th^ July to 5^th^ August 2020, and the third wave, conducted in Taiwan and Indonesia, were obtained between 21^st^ September to 14^th^ October 2020. The first wave countries were identified by the authors as key areas for obtaining a global perspective, and countries in subsequent waves were selected as local affiliates of the study sponsor with confirmed interest in the survey.

The surveys and data analyses were conducted in compliance with the Market Research Society Professional Code of Conduct and data protection laws, including the General Data Protection Regulation. No personal details of the participants were collected or stored.

### Patient and public involvement

The survey participants were members of the public. Study steering committee member and author M.B. has lived experience with heart failure and represents a patient advocacy group.

### Survey questions

The general public and policymaker survey questions were developed by members of the author team (A.C. and P.P.-H.). The aim of the general public survey was to gauge the public’s awareness and understanding of what HF is, in comparison to other common conditions; awareness and understanding of HF symptoms, risk factors and mortality; and views around hospital admissions in their country. The policymaker survey questions were developed to determine their awareness and understanding of HF, in comparison to other common conditions; awareness and understanding of the causes of hospital admissions in their country; and views on the sustainability of the healthcare system and healthcare budget priorities.

### Analysis

The number of participants who selected each response to the questions was calculated as a percentage of the total number of participants for that country, and of the total number of all respondents. For Likert scale questions where respondents indicated their level of agreement with a statement (strongly agree, slightly agree, neutral, slightly disagree, strongly disagree), the net total of ‘strongly agree’ and ‘slightly agree’ was calculated to assess the level of agreement.

## Results

### Demographics

The general public survey was completed by 26,272 respondents (52% female) from 13 countries (Table 1). Respondent age distribution was: 3503 (13%) were 18–24 years, 5492 (21%) were 25–34 years, 5329 (20%) were 35–44 years, 4356 (17%) were 45–54 years, 4456 (17%) were 55–64 years, and 3136 (12%) were ≥ 65 years. The policymaker survey was completed by 281 respondents from nine countries.

### Study outcomes

#### Awareness and understanding of HF and its symptoms

**Figure 1:**
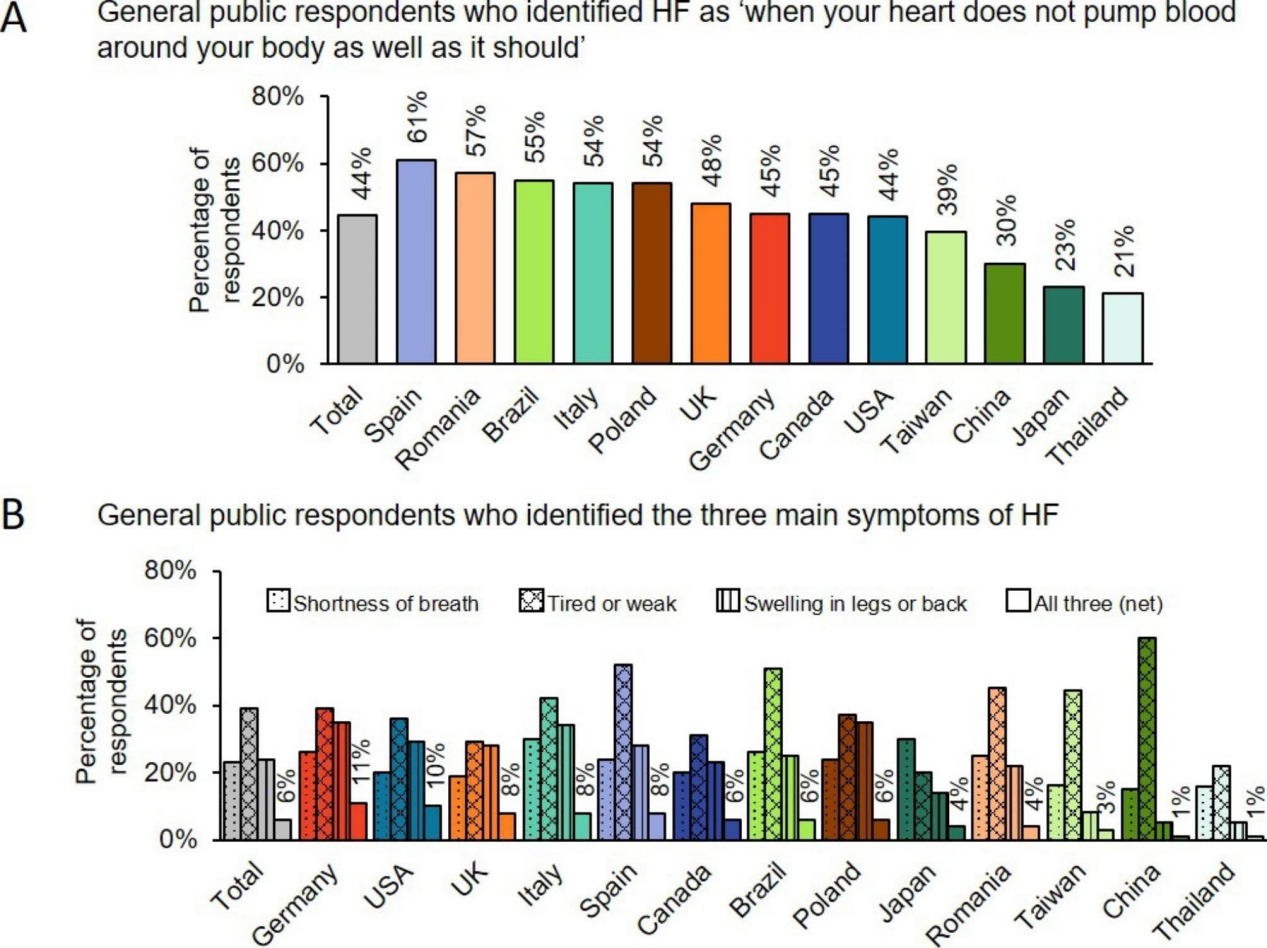
**Understanding of the definition of HF.** Percentage of general public respondents, by country, who correctly identified (A) the definition of HF, and (B) the three most common symptoms of HF. (A) Participants were asked ‘Which of the following statements do you think best describes HF? HF is when your heart does not pump blood around your body as well as it should; HF is when your heart stops beating; HF is when the supply of blood to the heart is suddenly blocked, usually by a blood clot; HF is a serious life-threatening condition that happens when the blood supply to part of the brain is cut off; HF is a gradual and natural weakness of the heart as a person ages; Don’t know’. (B) Presented as three questions (i–iii), participants were asked ‘If a person had (i) shortness of breath, (ii) feeling tired or weak, and (iii) swelling of the feet, ankles, legs, abdomen or in the small of your back, which condition would you think they had? Heart failure; Diabetes; Heart attack; Stroke; Asthma; Don’t know’. The results for each of the three questions are shown, together with the net total of respondents who selected ‘heart failure’ for all three symptoms. HF, heart failure

Globally, 99% of general public study participants were aware of HF, but only 44% identified that HF is ‘when your heart does not pump blood around your body as well as it should’ (Fig. 1A).[27] In Spain, the majority of respondents (61%) identified the correct definition, and just over half in Romania (57%), Brazil (55%), Italy (54%) and Poland (54%). In China (30%), Japan (23%) and Thailand (21%), a minority of respondents identified the correct definition. Fatigue was recognised as a major symptom of HF by 39% of respondents, but awareness of shortness of breath (22%) and leg swelling (22%) as the other two symptoms was lower. Nationally, the percentage of general public respondents who associated HF with all three major symptoms ranged from 11% in Germany to 1% in China and Thailand, with a global rate of 6% (Fig. 1B).

#### Awareness and understanding of HF risk factors and mortality

Under a quarter of public respondents were aware that the lifetime risk of developing HF is one in five,[20] (ranging from 36% in Canada to 14% in Germany; Supplementary Figure 1A). Over half of policymaker respondents in Italy (56%) and 43% in both Canada and Germany correctly identified the risk of developing HF; however, globally, overall awareness was 38% in this group.

Only 12% of general public respondents were aware of the high mortality rate in HF (Supplementary Figure 1B).[21] The countries with the highest proportion of general public respondents that correctly understood HF mortality were Thailand (20%), Taiwan (19%) and the USA (16%). Globally, 36% of general public respondents identified conditions such as diabetes, hypertension and coronary heart disease as the leading risk factors for developing HF (Supplementary Figure 2).[10] The highest proportion of respondents to correctly identify these risk factors was in Thailand (46%) and Poland (45%). In Japan, most respondents (31%) believed that smoking is the leading risk factor for developing HF.

#### General public views around hospital admissions in their country

**Figure 2:**
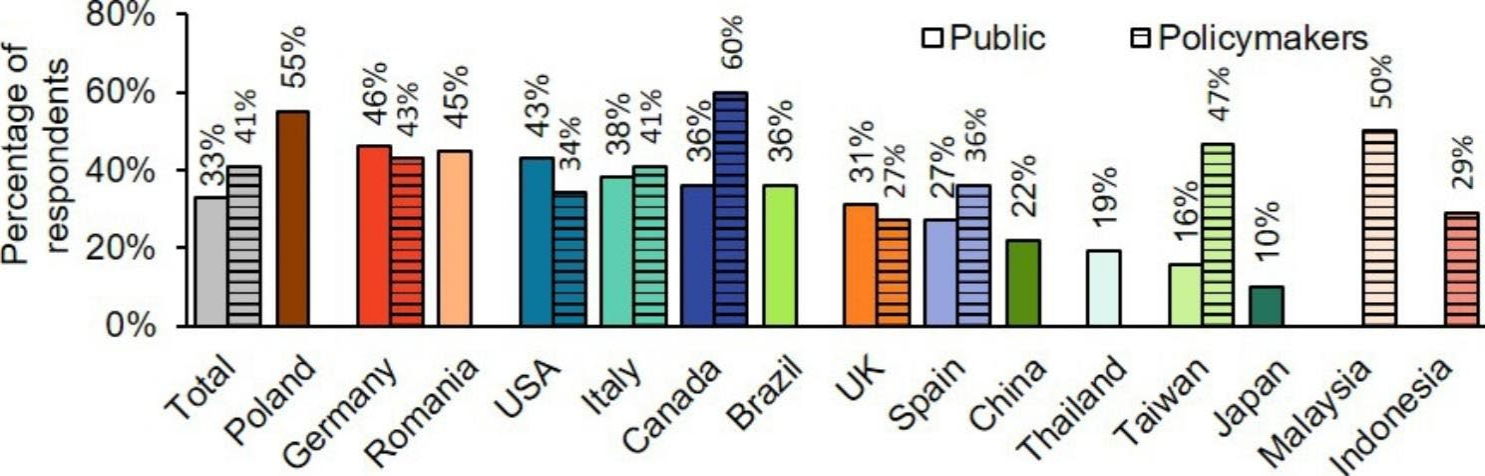
**Understanding of the impact of HF on healthcare systems.** Percentage of general public and policymaker respondents, by country, who identified HF as the leading reason for hospital admission in people over 65 years old. Participants were asked ‘What do you think is the number one reason people over 65 are admitted to hospital? Heart failure; Cancer; External causes (e.g. suicide, accidents); Respiratory disease (e.g. asthma, COPD); Chronic kidney disease; Alzheimer’s disease; Other; Don’t know’. COPD, chronic obstructive pulmonary disease; HF, heart failure

Only a third of public respondents correctly recognised that HF is the leading cause of hospitalisations for people over 65 years,[5, 28] with awareness ranging from 55% in Poland to 10% in Japan (Figure 2). Almost half of general public respondents agreed that there is a need to reduce hospital admissions in their country (Supplementary Figure 3A).

**Figure 3:**
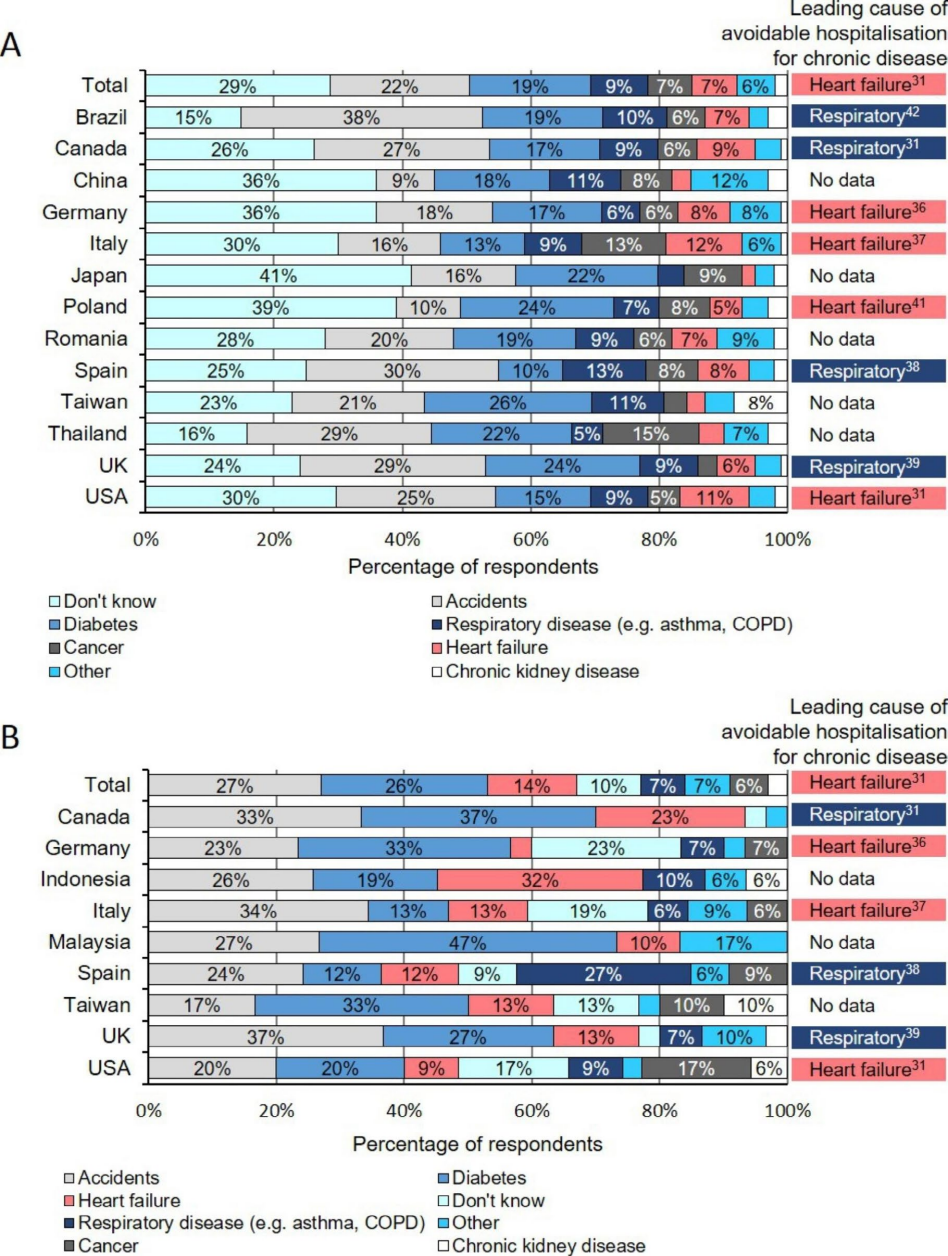
**Understanding of the leading cause of avoidable hospitalisations by country.** (A) General public and (B) policymaker responses, by country, to the question ‘What do you think is the number one reason for avoidable hospital admissions in your country?’. There is a lack of evidence comparing all causes of avoidable hospitalisations, but the leading cause of avoidable hospital admissions for chronic disease is listed to the right of the plots where these data could be identified.[29–36] Congestive HF causes more avoidable hospital admissions than diabetes and respiratory disease globally, and in Germany, Italy, Poland and the USA.[29, 30, 34, 35] In the UK, Spain, Brazil and Canada, respiratory disease causes more avoidable hospital admissions than congestive HF.[31, 32, 35, 36] Values below 5% are not labelled on the plots. HF, heart failure

A minority of total general public respondents (7%) selected HF as the leading reason for avoidable hospital admissions in their country; 12% of respondents in Italy and 11% in the USA selected this, compared with 3% in China and Taiwan, and 2% in Japan (Figure 3A). In the majority of countries, most general public respondents indicated that they did not know the leading reason for avoidable hospitalisations (Figure 3A).

#### Policymaker awareness and understanding

A total of 41% of policymaker respondents were aware that HF is the leading cause of hospitalisation for people over 65 years old, with 60% awareness among respondents from Canada, compared with 27% in the UK (Figure 2). The majority of policymaker respondents (58%) agreed that there is a need to reduce hospital admissions in their country, ranging from 77% in the USA to 38% in Italy (Supplementary Figure 3A). Only 4% of policymaker respondents were aware that up to 87% of government healthcare spend on HF is associated with hospitalisations,[11] ranging from 13% in Canada to no respondents in Italy, Germany, Malaysia and Taiwan (Supplementary Figure 3B).

Globally, only 14% of policymaker respondents selected HF as the leading cause of avoidable hospitalisations in their country, whereas 27% believed accidents to be the leading cause (Figure 3B). In Germany, only 3% of policymaker respondents indicated that HF is the leading cause of avoidable hospitalisations in their country.

**Figure 4:**
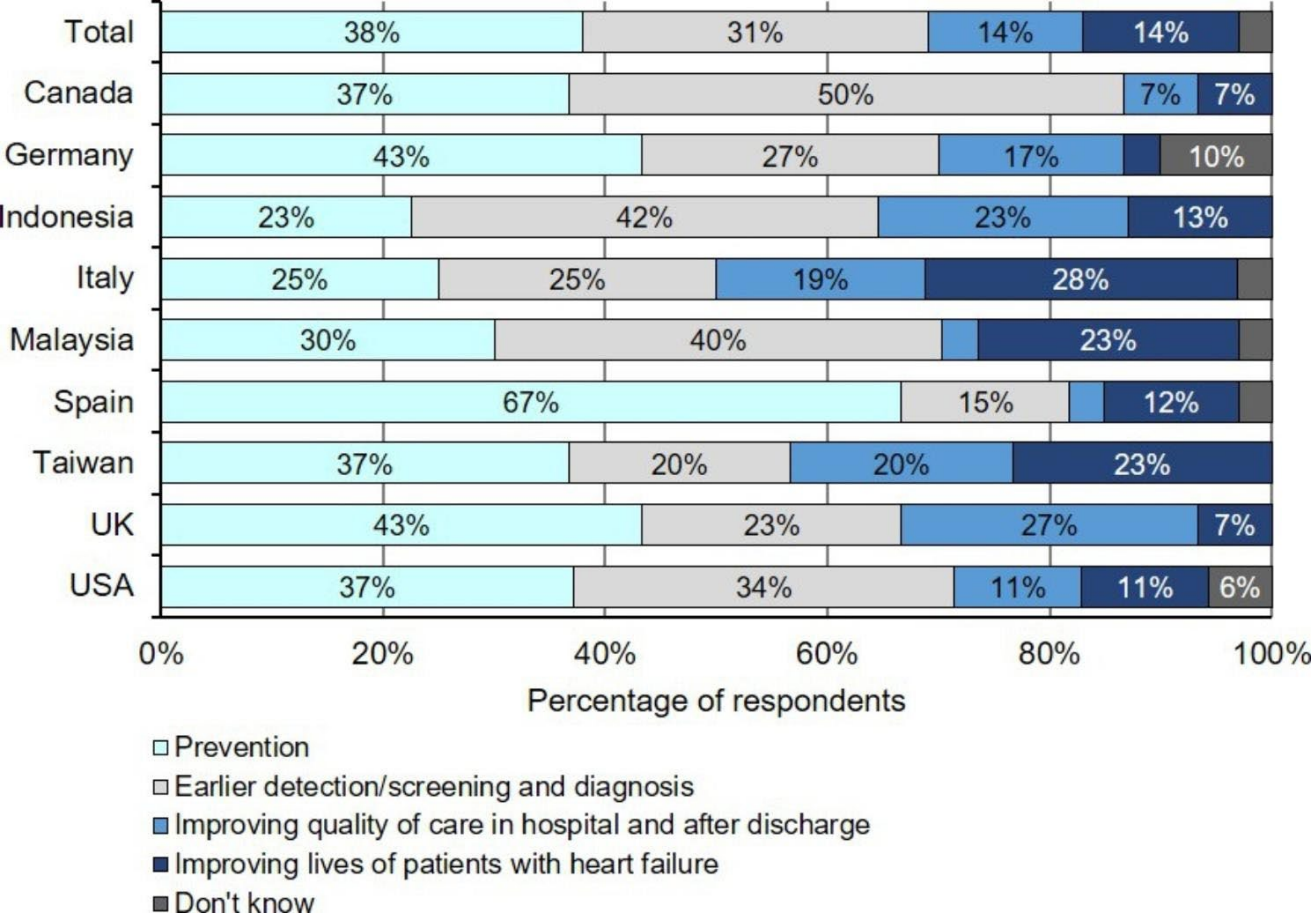
**Policymaker opinion on improvements that should be prioritised in the care of patients with HF.** Policymaker responses, by country, to the question ‘From what you know about HF and thinking about how patients are treated in the health system in your country, which areas should be prioritised for improvement?’. Values below 5% are not labelled on the plots. HF, heart failure

When asked about improvements that should be prioritised regarding the care of patients with HF, 38% of policymaker respondents selected ‘Prevention’ (Figure 4). This was greater than those who selected ‘Earlier detection, screening, and diagnosis’ (31%). Only 14% selected ‘Improving quality of care in hospital and after discharge’ or ‘Improving the lives of patients with HF’ as priority areas for improvement.

## Discussion

The findings of this survey indicate that large gaps exist in general public and policymaker understanding of HF. This mirrors an earlier statement from the Heart Failure Association of the European Society of Cardiology that there is a pressing need for public awareness programmes that explain the signs and symptoms of HF.[37] Our findings are consistent with previous studies in this area,[18, 19, 38, 39] extending prior literature with a wide global reach to include not only members of the general public, but also policymakers. A survey of the general public, conducted in Europe in 2005, showed that although 86% of respondents were aware of HF, only 3% could correctly identify the condition from a description of typical signs and symptoms. Moreover, only 29% considered the symptoms of HF as serious, while many of the respondents thought that HF was a normal consequence of ageing, and wrongly thought that modern medicine cannot prevent the development of HF.[18] Ten years later, similar results were reported for the general public in Slovenia.[38] A recent study analysing the knowledge of the general population on HF in Poland also showed that the majority of the participants reported a poor understanding of HF; however, this time symptoms of HF were correctly identified by the majority. It was noted that, approximately one-third of the respondents, with or without HF, believed that sport was not advisable for patients with HF, stressing the need for further awareness that physical activity can improve the prognosis of HF.[19] Furthermore, a study in Germany that took place over 8 years reported no improvement in HF awareness, despite several campaigns to improve HF awareness being held during this period. The study also reported age as factor that determined which types of information sources were used to obtain information about health and HF; in particular, younger respondents used the internet, whereas older participants relied more on printed and verbal media, and on their GP. This should be considered when planning future awareness activities.[39] The results of the current survey reveal that overall awareness and understanding of HF have improved, but fewer than half of respondents were able to accurately define the condition, and only 6% recognised all three cardinal clinical features of HF. Patterns of respondents recognising the definition and symptoms of HF were consistent across countries, although numbers were lower in the East Asia region than in Europe or the Americas.

Low awareness of HF among the general public and policymakers may come at significant opportunity cost. A lack of understanding of HF causes and symptoms may prevent people from seeking medical attention promptly, leading to large numbers of premature deaths.[18, 37] Currently, 80% of patients with HF are only diagnosed when hospitalised with severe, advanced symptoms despite presenting to general practitioners on several prior occasions.[40] HF manifests in multiple ways; as such, patients take various routes to seek healthcare. For example, patients with mild symptoms of HF may not see their GP, whereas severe, sudden onset of symptoms may lead to direct admission to the hospital through the emergency department, thereby bypassing the GP. Moreover, it was reported that often patients did not receive guideline-directed therapy or GPs did not follow guideline recommendations for the diagnosis of HF in the majority of patients who went on to receive an HF diagnosis. [40] Increased awareness among primary care physicians of the clinical and economic burden of HF and the benefits of early diagnosis may relieve some of the pressure that late diagnosis places on secondary care. Timely initiation of evidence-based treatments would improve patient outcomes and healthcare resource use.[41] It has also been reported that favourable illness perception is associated with better health outcomes, while unfavourable illness perception has been associated with worse outcomes. Sawyer and colleagues suggest that interventions targeting illness perception via a multifaceted approach, which includes behavioural, clinical, educational and psychosocial components, could improve health outcomes in patients with HF and prevent untimely readmission of such patients.[42].

This study is the first, to our knowledge, to provide detailed evidence for policymaker awareness of HF and priorities for care strategies. This respondent group indicated that prevention of HF is their first priority for improving the care of people living with the disease. While a vital goal of national strategies to address the burden of HF should be prevention, this will take time to achieve, and HF cohorts are likely to grow in the immediate future.[7] Policymaker understanding of the costs associated with HF was found to be low, which may confound efforts by advocates to demand that patients receive guideline-based care, supported by improved resourcing and political oversight. Accidents were identified by the majority of policymakers as the leading cause of avoidable hospital admissions, but data suggests that chronic diseases cause a higher proportion of avoidable admissions than accidents, and heart failure is the leading cause of avoidable hospital admissions for chronic disease globally.[35, 43] Patients with HF experience substantially higher hospital admission and readmission rates than age- and sex-matched controls.[44, 45] Despite recognising the need to reduce hospitalisations in general, many policymaker respondents underestimated the hospitalisation burden and government healthcare costs associated with HF. The safe reduction of hospital readmissions should be prioritised within national strategies as part of a sustainable approach to HF care.[13] Improving policymaker knowledge of the costs associated with HF and the number of avoidable hospital admissions would be a critical first step towards improved prioritisation of resources to reduce this growing burden. There is a lack of recent data on the social and economic costs of HF, and broader analyses of preventable causes of hospital admissions, which might contribute to low awareness of the burden of HF.[10, 20, 21] To accurately inform policy decisions, policymakers need to be informed by current and accurate data on the major direct and indirect cost drivers of HF.[46].

Despite admirable efforts by HF stakeholders, the findings of this study underscore a consequence of the historical absence of dedicated HF advocates in many countries over many years. Evidently, the scale and burden of HF upon our societies urgently demand renewed, persistent and voluminous efforts to explain the relevance of HF to decision-makers and elected officials. To gain maximum exposure for policy change and renewed investments, we believe that clear, evidence- and consensus-based arguments should be developed via close partnerships between stakeholders such as patient advocacy groups, clinical societies, academia, healthcare management, payers, industry and public policy non-governmental organisations. Ideally, this would be with political backing and funding, and be well aligned to national strategic plans in HF. Decision-makers and political commentators could benefit greatly by comprehensive national HF registries and periodic audits in HF to raise awareness of HF as a priority area for societal benefit. We further propose that future efforts should also be innovative and complement outreach to traditional decision-makers (such as ministries of health or government health agencies); HF should be highlighted as an area of relevance and opportunity across wider public policy themes. For example, focus could include societal inequalities in access to HF diagnosis, care and patient outcomes. HF as a field might also integrate well into the growing interest among high income countries in life science industrial innovation and expansion, as a future pillar of wealth generation in a globalised economy. Thus, increased awareness of HF could be accomplished by targeting basic information on HF and by using an adapted narrative for investment strategies, aimed at politicians that are devoted to wider economic, industrial, and societal topics, with roles at ministerial level. On a final note, the power of patient advocacy groups appears to be largely unharnessed, as compared with other disease areas like cancer. Efforts to upskill, grow and evolve national and local HF patient advocacy groups as a body of strong and vocal political representation will likely be critical.

There are several limitations of this study. The results are representative only of the countries and regions in which the survey was conducted, as differences in healthcare systems and provision of care in areas that were omitted in this study might have yielded different results. Also, the policymakers were not recruited from all countries where public surveys were conducted due to insufficient number of the established pool of contacts, and thus results should be interpreted accordingly. Representative of national demographics, the proportion of survey respondents >65 years of age was 12%, but the burden of HF is reportedly higher for this age group.[5, 28] The survey questions, while carefully designed, were not validated. When asked which condition they associated with shortness of breath, fatigue and leg swelling, respondents could select only one condition out of HF, diabetes, heart attack, stroke and asthma. Given that, for example, shortness of breath is also associated with asthma, respondent awareness that these symptoms are associated with HF may have been under-represented.

## Conclusion

The COVID-19 pandemic and its associated requirement for beds and capacity in hospitals have brought into stark relief the need to reduce avoidable hospital admissions through improved diagnosis and care. Results of this study show that large gaps remain in the understanding of HF, its symptoms, and its morbidity and mortality burden among both policymakers and the general public across countries of different continents. Improvement of the knowledge of HF among the general public may lead to the more rapid seeking of medical attention, which could reduce the stress on patients, caregivers and healthcare systems. Moreover, awareness of policymakers of the burden and cost associated with HF, and the number of avoidable hospital admissions, could lead to improved public health policies. This shows the urgent need for greater advocacy and awareness-raising efforts in HF, alongside greater access to key elements of effective care and management; however, how to accomplish this most effectively remains to be determined.

## Electronic supplementary material

Below is the link to the electronic supplementary material.

Supplemental Figure 1. Understanding of the health impact of HF

## Data Availability

The datasets generated and/or analysed during the current study are not yet publicly available as a suitable data sharing platform has yet to be identified by the study sponsor, but are available from the corresponding author on reasonable request.
