# Supplementary material for: Identification of urgent gaps in public and policymaker knowledge of heart failure: Results of a global survey
Source: BMC Public Health. 2023 May 30;23:1023. doi: 10.1186/s12889-023-15405-4 (PMC10227786; doi:10.1186/s12889-023-15405-4)
Supplement: Supplementary file 1 — Supplemental Figure 1. Understanding of the health impact of HF [file 12889_2023_15405_MOESM1_ESM.pdf]

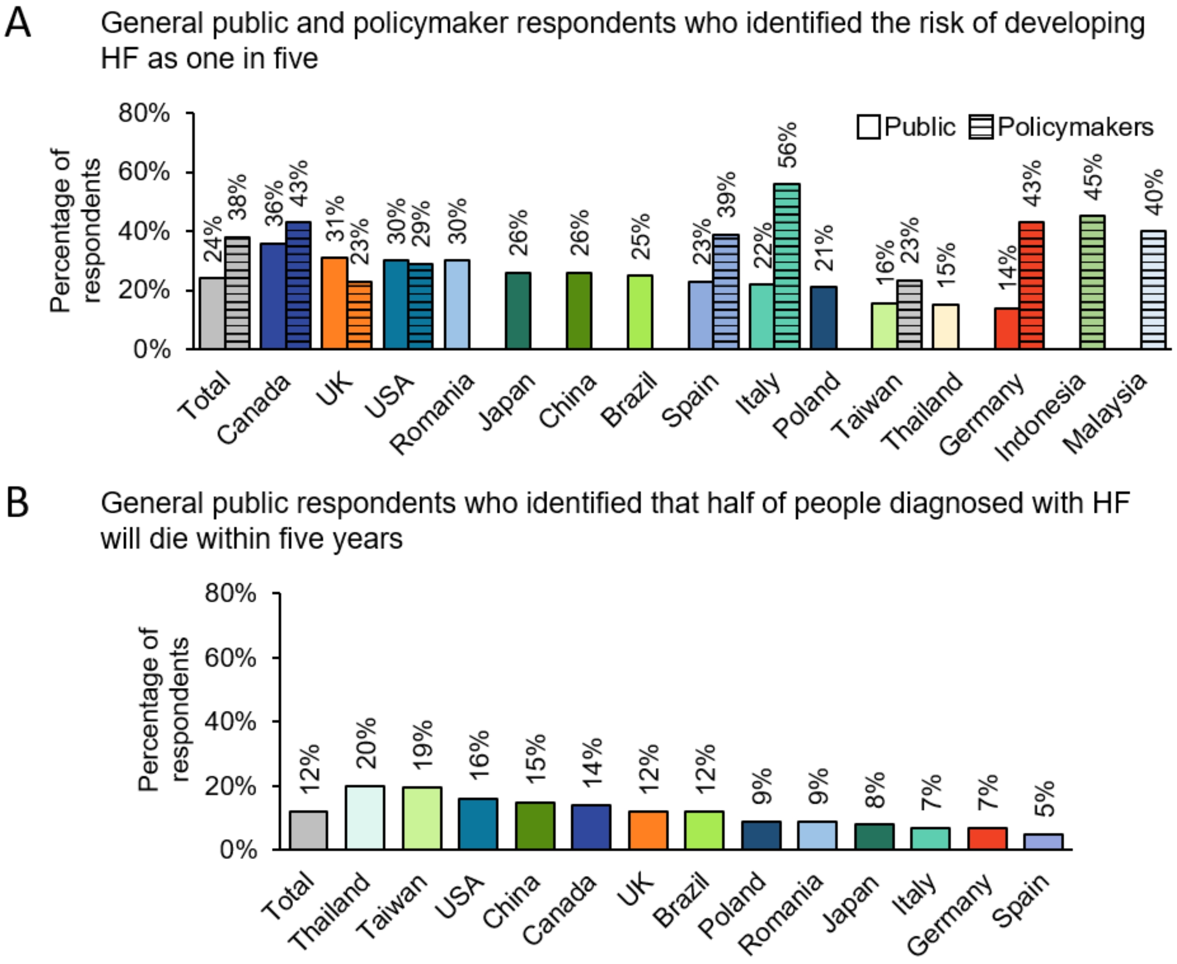

Supplemental Figure 1. Understanding of the health impact of HF. (A) Percentage of general public and policymaker respondents, by country, who correctly identified the risk of developing HF. Participants were asked 'What do you think is a person's risk of developing HF? 1 in 50; 1 in 20; 1 in 5; 1 in 2; Don't know'. (B) Percentage of general public respondents, by country, who correctly identified that half of people diagnosed with HF die within five years of their diagnosis. Participants were asked 'Which of the following statements about HF do you think is true? Half of people diagnosed with HF die within five years of their diagnosis; Half of people diagnosed with HF die within 10 years of their diagnosis; Most people do not die as a result of HF, but usually old age or another condition; Don't know'. HF, heart failure.

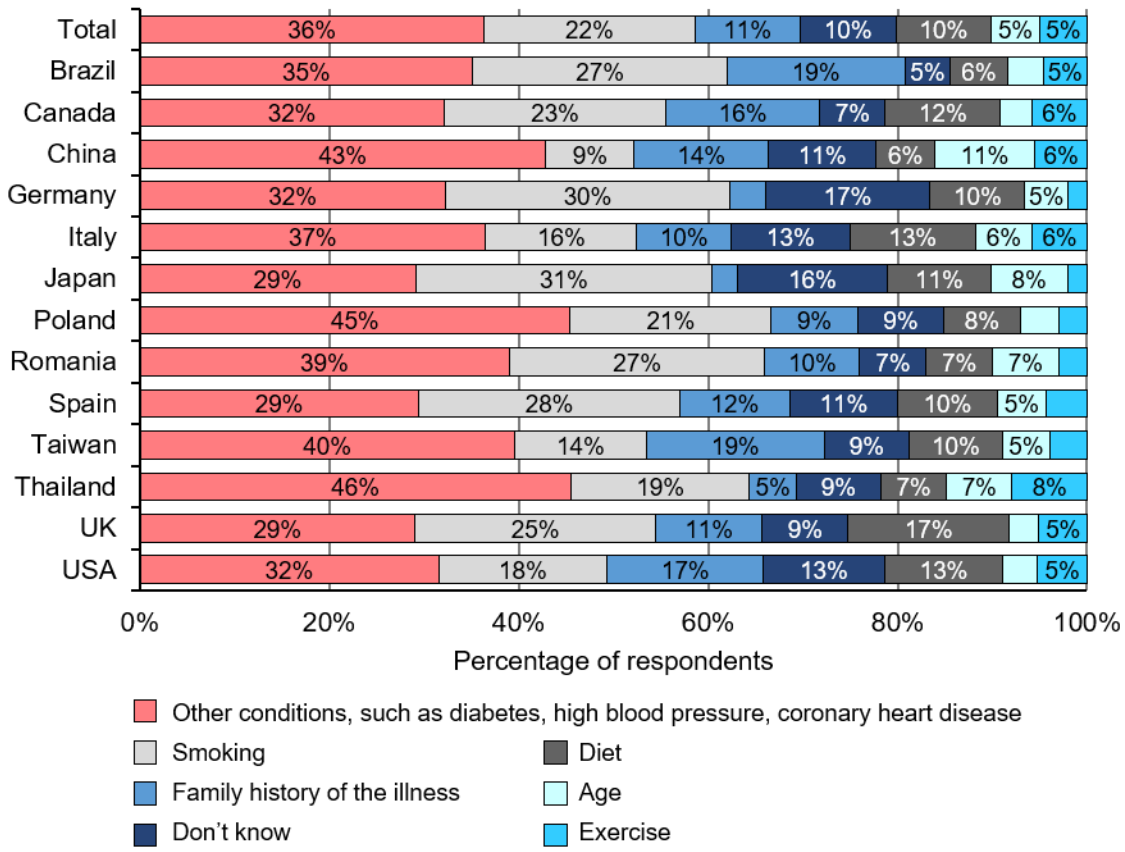

Supplemental Figure 2. General public understanding, by country, of the leading risk factors for developing HF. General public responses, by country, to the question 'Which of the following do you think has the most impact on a person's risk of developing HF?' Globally, diabetes, high blood pressure and coronary heart disease, as well as smoking, are recognised as leading risk factors for developing HF, although this risk varies by sex, age, ethnicity, comorbidities and environment.(1) Values below 5% are not labelled on the plot. HF, heart failure.

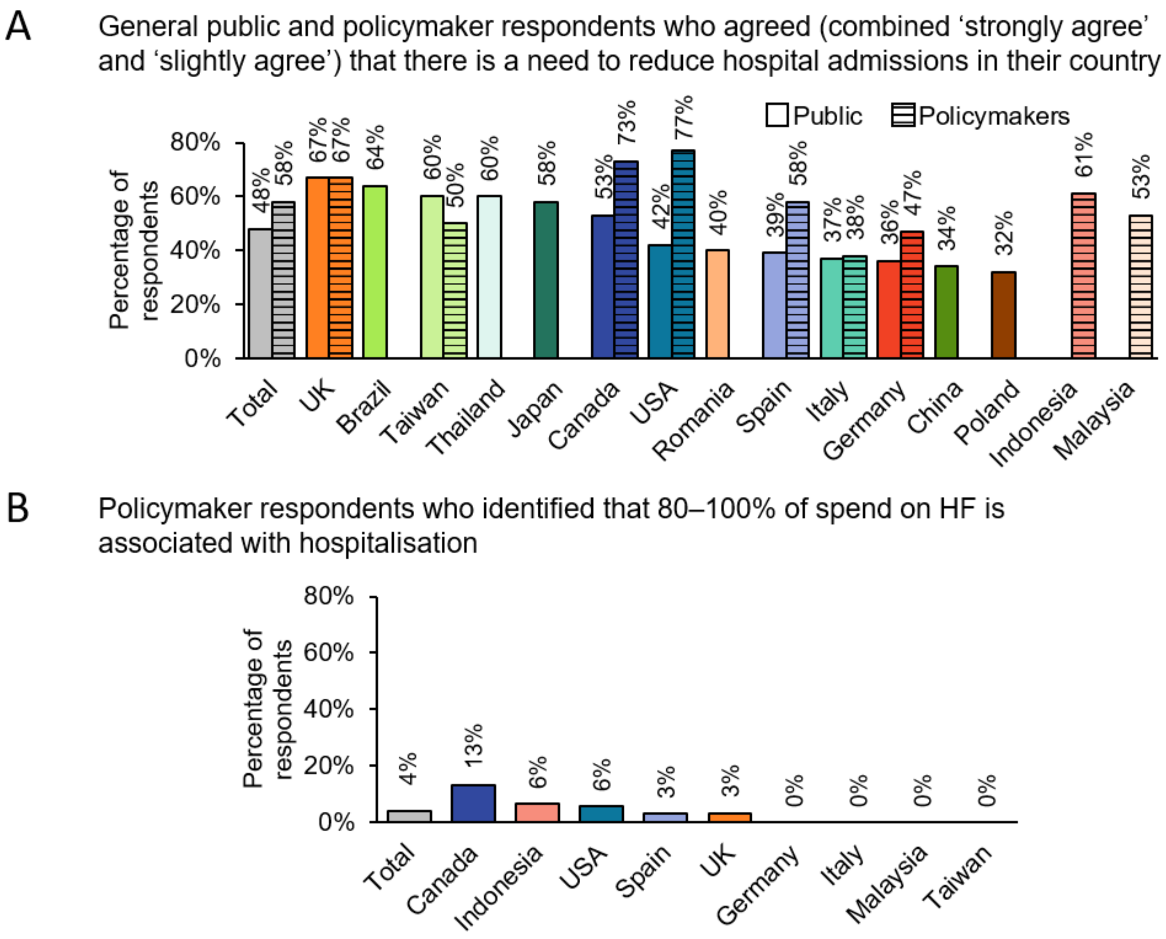

Supplemental Figure 3. Understanding of the impact of HF on healthcare systems.

(A) Percentage of general public and policymaker respondents, by country, who agreed that there is a need to reduce hospital admissions in their country. Participants were asked 'To what extent do you agree or disagree that there is a need to reduce hospital admissions in your country? Strongly agree; Slightly agree; Neutral; Slightly disagree; Strongly disagree; Don't know'. (B) Percentage of policymaker respondents, by country, who identified that up to 87% of government spend on HF is associated with hospitalisations. Participants were asked 'Governments spend 1–2% of their health system budget on HF. Globally, this amounts to around USD 108 billion spent annually on HF (2012 data). Approximately what proportion of this spend do you think is associated with hospital admissions? 80–100%; 60–79%; 40–59%; 20–39%; 0–19%; Don't know'. HF, heart failure.

Supplemental Appendix 1. General public survey questions as presented to respondents on the YouGov platform (2)

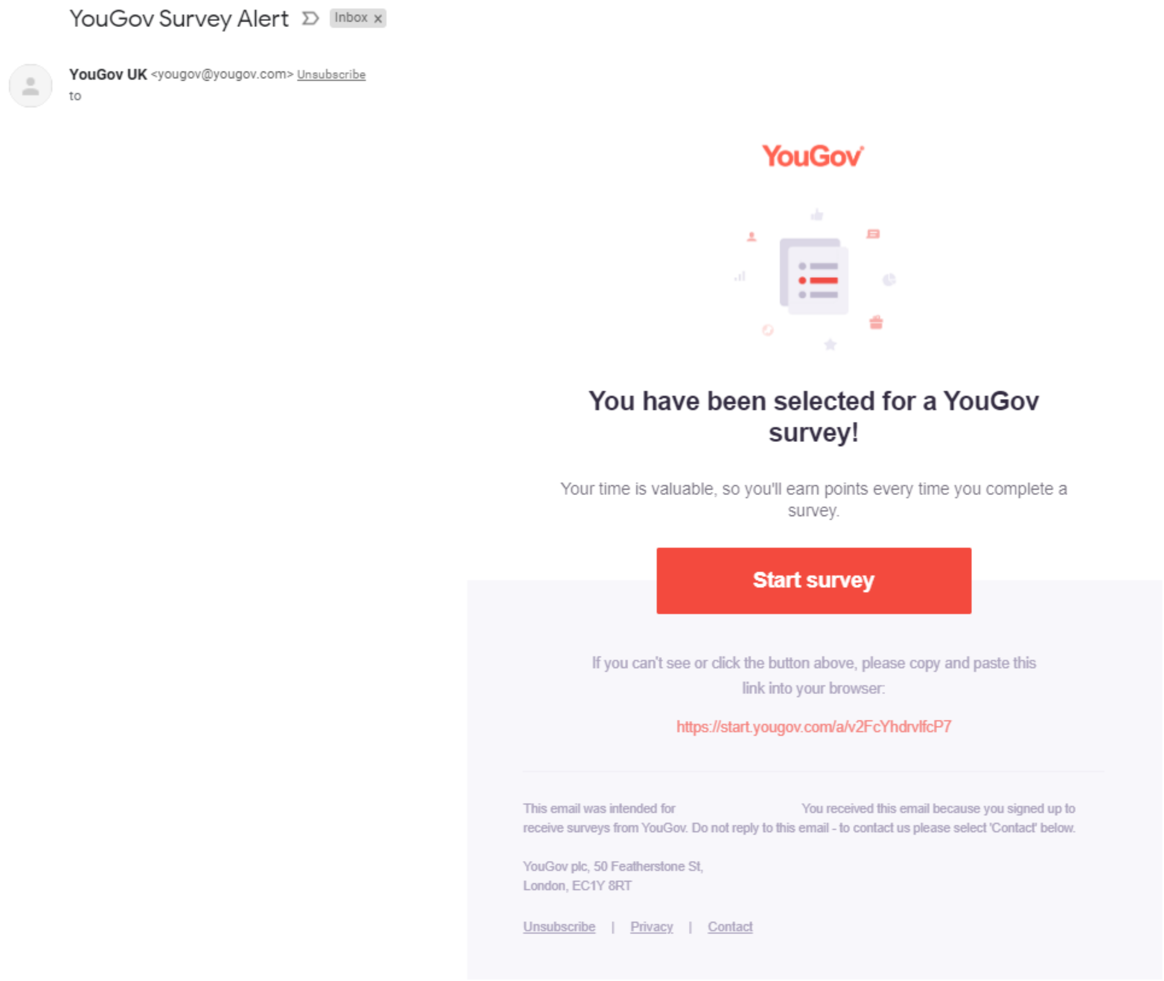
YouGov Survey Alert Inbox x

YouGov UK <yougov@yougov.com> Unsubscribe
to

YouGov

You have been selected for a YouGov survey!

Your time is valuable, so you'll earn points every time you complete a survey.

Start survey

If you can't see or click the button above, please copy and paste this link into your browser:

https://start.yougov.com/a/v2FcYhdrvlfcP7

This email was intended for You received this email because you signed up to receive surveys from YouGov. Do not reply to this email - to contact us please select 'Contact' below.

YouGov plc, 50 Featherstone St,
London, EC1Y 8RT

Unsubscribe | Privacy | Contact

YouGov

Have you personally ever been diagnosed by a doctor as having any of the following conditions? *Please select all that apply.*

- Cancer
- Heart Failure
- Diabetes
- Chronic kidney disease
- Respiratory diseases (e.g. asthma, COPD)
- None of the above

>

Z4-22242 | January 2020
Expiry: 29 March 2020

SCREEN OUT IF SELECTED 'HEART FAILURE'

RESPONDENT WILL NOT PROGRESS ANY FURTHER IN THIS SURVEY AND WILL INSTEAD BE FED BACK TO THE YOUGOV ECOSYSTEM TO COMPLETE ANOTHER SURVEY. FROM THIS POINT THEY WILL NOT BE ATTACHED TO THE ASTRAZENECA SURVEY IN ANY WAY.

YouGov

Welcome to today's survey!

The purpose of this survey is to get a better understanding of the public's perception of health conditions, awareness of symptoms and how certain conditions differ from other illnesses.

We have tested the survey and found that, on average it takes 8-10 minutes to complete. This time may vary depending on factors such as, your Internet connection speed and the answers you give.

Your YouGov account will be credited with 50 points for completing the survey.

Please be assured that this survey conducted on behalf of our client, a pharmaceutical company, is completely confidential and complies with the Market Research Society Professional Code of Conduct and data protection laws, including General Data Protection Regulation (GDPR). The answers you provide will not be identified individually and will be compiled together and analysed as a group, so your responses will remain fully anonymous. Your information will only be used for market research and will not be passed to any other organisation without your permission. By entering the survey, you consent to the collection of this information.

You have the right to withdraw from the survey at any time by closing your browser window.

Please click the forward button to continue.

>

Z4-22242 | January 2020

YouGov

What do you think is the leading cause of death in your country? *Please select one answer.*

- External causes (e.g. suicide, accidents)
- Chronic Kidney Disease
- Respiratory diseases (e.g. asthma, COPD)
- Cardiovascular disease
- Cancer
- Other
- Don't know

Z4-22242 | January 2020

YouGov

What do you think is the number one reason people over 65 are admitted to hospital? *Please select one answer.*

- External causes (e.g. suicide, accidents)
- Chronic Kidney Disease
- Heart failure
- Alzheimer's Disease
- Respiratory diseases (e.g. asthma, COPD)
- Cancer
- Other
- Don't know

Z4-22242 | January 2020

YouGov

How much, if anything, do you know about each of the following conditions? *Please select one answer per condition.*

	A lot	A fair amount	Only a little	Hardly anything	Never heard of	Don't know
Heart Failure	○	○	○	○	○	○
Cancer	○	○	○	○	○	○
Alzheimer's Disease	○	○	○	○	○	○
Diabetes	○	○	○	○	○	○
Chronic Kidney Disease	○	○	○	○	○	○
Respiratory diseases (e.g. asthma, COPD)	○	○	○	○	○	○

Z4-22242 | January 2020

YouGov

Do you personally know anyone who has been diagnosed with each of the following conditions? *Please select all answers that apply for each condition.*

	Yes – member of family (child, parent, sibling or spouse)	Yes – friend, neighbour, colleague, co-worker	No	Don't know
Cancer	☐	☐	☐	☐
Heart Failure	☐	☐	☐	☐
Diabetes	☐	☐	☐	☐
Respiratory diseases (e.g. asthma, COPD)	☐	☐	☐	☐
Chronic Kidney Disease	☐	☐	☐	☐

Z4-22242 | January 2020

YouGov

What do you think is the number one reason for avoidable hospital admissions in your country? *Please select one answer.*

- Respiratory diseases (e.g. asthma, COPD)
- Heart Failure
- Diabetes
- Chronic Kidney Disease
- Accidents
- Cancer
- Other
- Don't know

Z4-22242 | January 2020

YouGov

To what extent do you agree or disagree that there is a need to reduce hospital admissions in your country? *Please select one answer.*

- Strongly agree
- Slightly agree
- Neutral
- Slightly disagree
- Strongly disagree
- Don't know

Z4-22242 | January 2020

YouGov

If a person had the following symptoms, which condition would you think they had? *Please select one answer per set of symptoms.*

	Heart failure	Diabetes	Heart attack	Stroke	Asthma	Don't know
Swelling of the feet, ankles, legs, abdomen, or in the small of your back	○	○	○	○	○	○
Shortness of breath when you are resting or being active	○	○	○	○	○	○
Feeling unusually tired or weak	○	○	○	○	○	○

Z4-22242 | January 2020

YouGov

What do you think is a person's risk of developing heart failure? *Please select one answer.*

- 1 in 2
- 1 in 50
- 1 in 20
- 1 in 5
- Don't know

Z4-22242 | January 2020

YouGov

Which of the following statements about heart failure do you think is true? *Please select one answer.*

Before a diagnosis with heart failure, people may have symptomns...

- And any heart damage to their heart can be reversed
- But no damage will have been done to their heart
- And irreversible damage can be done to their heart
- Don't know

Z4-22242 | January 2020

YouGov

Which of the following statements about heart failure do you think is true? *Please select one answer.*

- Heart failure can be cured
- Heart failure cannot be treated
- Heart failure can be treated
- Don't know

Z4-22242 | January 2020

YouGov

Which of the following statements about heart failure do you think is true? *Please select one answer.*

- Heart failure can affect people of all ages
- Only older people develop heart failure
- Don't know

Z4-22242 | January 2020

YouGov

Which of the following statements about heart failure do you think is true? *Please select one answer.*

- Half of people diagnosed with heart failure die within 5 years of their diagnosis
- Half of people diagnosed with heart failure die within 10 years of their diagnosis
- Most people do not die as a result of heart failure, but usually old age or another condition
- Don't know

Z4-22242 | January 2020

YouGov

Which of the following statements do you think best describes heart failure? *Please select one answer.*

- Heart failure is a gradual and natural weakness of the heart as a person ages
- Heart failure is when the supply of blood to the heart is suddenly blocked, usually by a blood clot
- Heart failure is when your heart stops beating
- Heart failure is when your heart does not pump blood around your body as well as it should
- Heart failure is a serious life-threatening condition that happens when the bloody supply to part of the brain is cut off
- Don't know

Z4-22242 | January 2020

YouGov

Which of the following do you think has the most impact on a person's risk of developing heart failure? *Please select one answer.*

- Exercise
- Family history of the illness
- Smoking
- Age
- Diet
- Other conditions, such as diabetes, high blood pressure, coronary heart disease
- Don't know

Z4-22242 | January 2020

YouGov

This survey was commissioned by AstraZeneca. No personal data will be shared with AstraZeneca.

If you or anyone you know is experiencing any of the symptoms discussed in this survey then please seek the advice of a healthcare professional as they are best placed to advise.

Z4-22242 | January 2020

YouGov

Thank you for taking part, your input is hugely appreciated.

Z4-22242 | January 2020

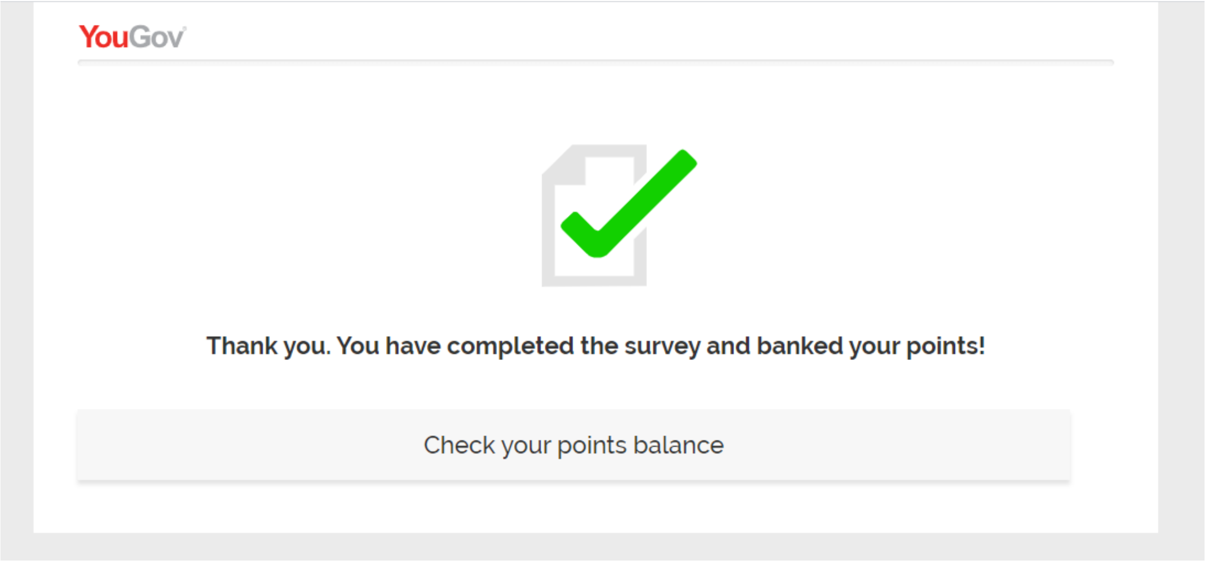
YouGov
Thank you. You have completed the survey and banked your points!
Check your points balance

Supplemental Appendix 2. Policymaker survey questions as presented to respondents on the YouGov platform (2)

Dear…

YouGov has been commissioned to get a better understanding of perceptions of health conditions, awareness of symptoms and how certain conditions differ from other illnesses.

This survey is conducted on behalf of our client, a pharmaceutical company. They are particularly interested in the perceptions of Politicians and Senior Policy Advisors in several countries.

The answers you provide will be combined with all other respondents across several countries and will be randomised and anonymously collated. Your data will be handled with strict privacy and stored according to GDPR requirements.

We would appreciate your participation in this important study. It should require only 10 minutes of your time.

SURVEY LINK

Your participation is important to us and your opinion is highly valued for this important research.

If possible, please try to complete this survey within the next 2 or 3 days from the date this e-mail is received.

Thank you for your participation.

Z4-22241 | January 2020

Expiry: 29 March 2020

YouGov

Have you personally ever been diagnosed by a doctor as having any of the following conditions? *Please select all that apply.*

- Chronic kidney disease
- Heart Failure
- Diabetes
- Respiratory diseases (e.g. asthma, COPD)
- Cancer
- None of the above

Z4-22241 | January 2020
Expiry: 29 March 2020

SCREEN OUT IF SELECTED 'HEART FAILURE'

YouGov

Thank you for participating in the survey. Unfortunately, you have not qualified for this survey.

I do hope we can call on your services again in the future.

Z4-22241 | January 2020

YouGov

Welcome to today's survey!

The purpose of this survey is to get a better understanding of politician and policymakers' perception of health conditions, awareness of symptoms and how certain conditions differ from other illnesses.

We have tested the survey and found that, on average it takes 8-10 minutes to complete. This time may vary depending on factors such as, your Internet connection speed and the answers you give.

Please be assured that this survey conducted on behalf of our client, a pharmaceutical company, is completely confidential and complies with the Market Research Society Professional Code of Conduct and data protection laws, including General Data Protection Regulation (GDPR). The answers you provide will not be identified individually and will be compiled together and analysed as a group, so your responses will remain fully anonymous. Your information will only be used for market research and will not be passed to any other organisation without your permission. By entering the survey, you consent to the collection of this information.

You have the right to withdraw from the survey at any time by closing your browser window.

Please click the forward button to continue.

Z4-22241 | January 2020

YouGov

What do you think is the leading cause of death in your country? *Please select one answer.*

- Cardiovascular disease
- Cancer
- Respiratory diseases (e.g. asthma, COPD)
- Chronic Kidney Disease
- External causes (e.g. suicide, accidents)
- Other
- Don't know

Z4-22241 | January 2020

YouGov

What do you think is the number one reason people over 65 are admitted to hospital? *Please select one answer.*

- Heart failure
- Alzheimer's Disease
- Cancer
- External causes (e.g. suicide, accidents)
- Respiratory diseases (e.g. asthma, COPD)
- Chronic Kidney Disease
- Other
- Don't know

Z4-22241 | January 2020

YouGov

How much, if anything, do you know about each of the following conditions? *Please select one answer per condition.*

	A lot	A fair amount	Only a little	Hardly anything	Never heard of	Don't know
Cancer	○	○	○	○	○	○
Respiratory diseases (e.g. asthma, COPD)	○	○	○	○	○	○
Chronic Kidney Disease	○	○	○	○	○	○
Diabetes	○	○	○	○	○	○
Heart Failure	○	○	○	○	○	○
Alzheimer's Disease	○	○	○	○	○	○

›

Z4-22241 | January 2020

YouGov

Do you personally know anyone who has been diagnosed with each of the following conditions? *Please select all answers that apply for each condition.*

	Yes – member of family (child, parent, sibling or spouse)	Yes – friend, neighbour, colleague, co-worker	No	Don't know
Diabetes	☐	☐	☐	☐
Heart Failure	☐	☐	☐	☐
Respiratory diseases (e.g. asthma, COPD)	☐	☐	☐	☐
Chronic Kidney Disease	☐	☐	☐	☐
Cancer	☐	☐	☐	☐

›

Z4-22241 | January 2020

YouGov

What do you think is the number one reason for avoidable hospital admissions in your country? *Please select one answer.*

- Respiratory diseases (e.g. asthma, COPD)
- Accidents
- Chronic Kidney Disease
- Diabetes
- Cancer
- Heart Failure
- Other
- Don't know

Z4-22241 | January 2020

YouGov

To what extent do you agree or disagree that there is a need to reduce hospital admissions in your country? *Please select one answer.*

- Strongly agree
- Slightly agree
- Neutral
- Slightly disagree
- Strongly disagree
- Don't know

Z4-22241 | January 2020

YouGov

What do you think is a person's risk of developing heart failure? *Please select one answer.*

- 1 in 2
- 1 in 20
- 1 in 50
- 1 in 5
- Don't know

Z4-22241 | January 2020

YouGov

How sustainable, if at all, do you think the health care system is in your country?

- Extremely sustainable
- Very sustainable
- Somewhat sustainable
- Not so sustainable
- Not at all sustainable
- Don't know

Z4-22241 | January 2020

YouGov

Governments spend 1-2 per cent of their health system budget on heart failure. Globally, this amounts to around $108 billion spent annually on heart failure (2012 data). Approximately what proportion of this spend do you think is associated with hospital admissions? *Please select one answer.*

- 80-100%
- 60–79%
- 40-59%
- 20–39%
- 0–19%
- Don't know

Z4-22241 | January 2020

YouGov

From what you know about heart failure and thinking about how patients are treated in the health system in your country, which areas should be prioritised for improvement? *Please select one answer.*

- Earlier detection/screening and diagnosis
- Don't know
- Improving quality of care in hospital and after discharge
- Prevention
- Improving lives of patients with heart failure

Z4-22241 | January 2020

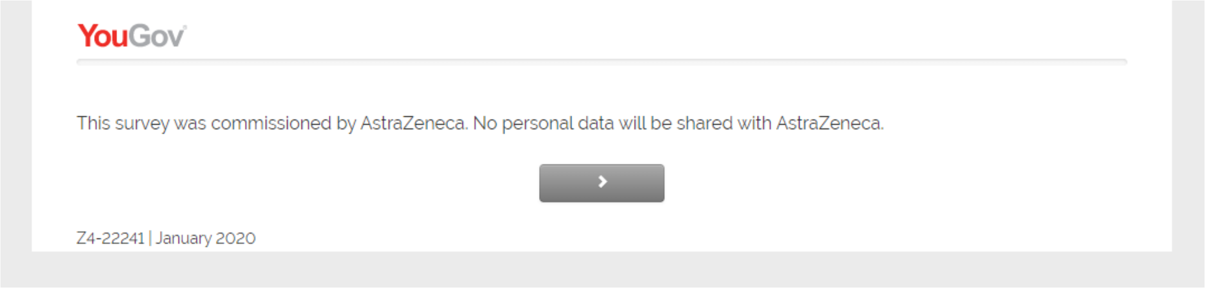

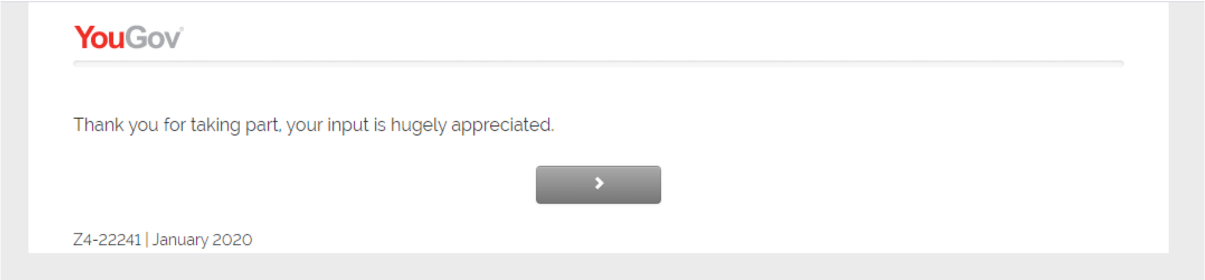

References

1. Ziaeian B, Fonarow GC. Epidemiology and aetiology of heart failure. Nature reviews Cardiology. 2016;13(6):368–78
2. YouGov. Available from: https://yougov.co.uk/ (Accessed January 2023)
